# Allosteric effects of *E. coli* SSB and RecR proteins on RecO protein binding to DNA

**DOI:** 10.1093/nar/gkad084

**Published:** 2023-02-20

**Authors:** Min Kyung Shinn, Sumit K Chaturvedi, Alexander G Kozlov, Timothy M Lohman

**Affiliations:** Department of Biochemistry and Molecular Biophysics, Washington University School of Medicine, St. Louis, MO 63110, USA; Department of Biomedical Engineering, Washington University in St. Louis, St. Louis, MO 63130, USA; Center for Biomolecular Condensates (CBC), Washington University in St. Louis, St. Louis, MO 63130, USA; Department of Biochemistry and Molecular Biophysics, Washington University School of Medicine, St. Louis, MO 63110, USA; Department of Biophysics, University of Delhi South Campus, New Delhi 110021, India; Department of Biochemistry and Molecular Biophysics, Washington University School of Medicine, St. Louis, MO 63110, USA; Department of Biochemistry and Molecular Biophysics, Washington University School of Medicine, St. Louis, MO 63110, USA

## Abstract

*Escherichia coli* single stranded (ss) DNA binding protein (SSB) plays essential roles in DNA maintenance. It binds ssDNA with high affinity through its N-terminal DNA binding core and recruits at least 17 different SSB interacting proteins (SIPs) that are involved in DNA replication, recombination, and repair via its nine amino acid acidic tip (SSB-Ct). *E. coli* RecO, a SIP, is an essential recombination mediator protein in the RecF pathway of DNA repair that binds ssDNA and forms a complex with *E. coli* RecR protein. Here, we report ssDNA binding studies of RecO and the effects of a 15 amino acid peptide containing the SSB-Ct monitored by light scattering, confocal microscope imaging, and analytical ultracentrifugation (AUC). We find that one RecO monomer can bind the oligodeoxythymidylate, (dT)_15_, while two RecO monomers can bind (dT)_35_ in the presence of the SSB-Ct peptide. When RecO is in molar excess over ssDNA, large RecO–ssDNA aggregates occur that form with higher propensity on ssDNA of increasing length. Binding of RecO to the SSB-Ct peptide inhibits RecO–ssDNA aggregation. RecOR complexes can bind ssDNA via RecO, but aggregation is suppressed even in the absence of the SSB-Ct peptide, demonstrating an allosteric effect of RecR on RecO binding to ssDNA. Under conditions where RecO binds ssDNA but does not form aggregates, SSB-Ct binding enhances the affinity of RecO for ssDNA. For RecOR complexes bound to ssDNA, we also observe a shift in RecOR complex equilibrium towards a RecR_4_O complex upon binding SSB-Ct. These results suggest a mechanism by which SSB recruits RecOR to facilitate loading of RecA onto ssDNA gaps.

## INTRODUCTION

*Escherichia coli* single stranded (ss) DNA binding (SSB) protein is a functional homo-tetramer (1,2) with each subunit comprised of two domains. The N-terminal domain (residues 1–112) binds non-specifically to ssDNA with high affinity (3–6). The C-terminal domain of SSB (SSB-Ct) (residues 113–177) consists of an intrinsically disordered linker (IDL) (residues 113–168) and the last nine amino acids (residues 169–177, MDFDDDIPF), termed the acidic tip. In bacteria, whereas the N-terminal DNA binding domains (DBD) are highly conserved, the IDL can vary in length from 25 to 125 amino acids, although none are highly charged.

*Escherichia coli* SSB protein binds polymeric ssDNA in multiple binding modes, depending on solution conditions (3–6).Two of the major binding modes are (SSB)_35_ and (SSB)_65_, where the subscripts denote the average number of nucleotides occluded per SSB tetramer (7–9). In the (SSB)_35_ mode, favored at high SSB to DNA ratios and low monovalent salt concentrations (<10 mM NaCl), 35 nucleotides interact with an average of two of the four subunits with unlimited cooperativity between nearest neighbor tetramers so that long protein clusters can form (9–12). In the (SSB)_65_ mode, favored at higher monovalent (>200 mM NaCl) and divalent (10 mM MgCl_2_) salt concentrations, 65 nucleotides interact with and wrap around all four subunits of SSB with cooperativity that limits clustering to dimers of tetramers (7,8,10–17). A non-nearest neighbor cooperativity, resulting in collapse of the ssDNA, can also occur at low [NaCl] and is also promoted by high acetate or glutamate concentrations in the physiological range (18–21). The *E. coli* SSB IDL is essential for all cooperative SSB-ssDNA interactions (18–20,22).

The acidic tips of the SSB-Ct act as a hub to recruit at least 17 proteins referred to as SSB interacting proteins (SIPs) (23), that are involved in DNA recombination (24–36), replication (37–41), replication restart (42–45), and repair (46–54). Unlike the IDL, the acidic tip is highly conserved in bacteria with the last two residues (Pro and Phe) being the most conserved. Mutation of the penultimate proline to serine or a deletion of the tip region disrupts SSB-SIP interactions (55,56). SSB-Ct acidic tip binds to different SIPs with specificity (55,57). The IDL region does not contribute to SIP binding (55,57). Up to four SIPs can bind to the four SSB-Ct within the SSB tetramer, and the acidic tips can also compete with DNA for binding to the DBDs. In full length SSBs, there may be additional interactions between SIPs and the DBDs (57).

*Escherichia coli* RecO and RecR are essential recombination mediator proteins (RMPs) in the RecF pathway that is primarily involved in repair of single stranded DNA gaps (58–63) but also plays a secondary role in double strand breaks (64,65). RecO binds to both ss and dsDNA and facilitates the annealing of complementary DNA strands (31,66). A crystal structure of RecO shows the two C-terminal residues of SSB-Ct (Pro and Phe) bound in a hydrophobic pocket of the central alpha helical region, similar to ExoI and RecQ (36,49,67). *E. coli* RecR, exists in a pH-dependent dimer-tetramer equilibrium and can form two species of protein complexes with RecO—RecR_4_O and RecR_4_O_2_—depending on the molar ratio of the two proteins (68). The main role of RecO, together with RecR, is to displace SSB molecules that are tightly bound to ssDNA and load RecA protein filaments onto ssDNA to initiate homologous recombination (33,69–76). *E. coli* RecO is a SIP (32,55,57), and a proposed mechanism for the loading of RecA onto ssDNA by RecOR suggests that RecO is recruited by the SSB-Ct through a direct interaction (77). However, the details of the interactions between the components of the RecOR pathway, RecO, RecR, SSB and ssDNA, and their stoichiometries are still unclear.

Binding of SIPs to the SSB-Ct was initially viewed only as a means to tether the SIP to SSB in order to facilitate its binding to DNA; however, it has been shown that SSB-Ct binding to at least some SIPs can exert an allosteric effect on SIP activities. SSB-Ct binding has a stimulatory effect on RecQ helicase activity (34,78). SSB-Ct peptide also stimulates ATP hydrolysis by *E. coli* RadD (54), a protein implicated in double strand (ds) break repair (79,80). Thus, the SSB-Ct may affect the properties of other SIPs (34,54,78). We have previously demonstrated an allosteric effect of an SSB-Ct peptide on the interaction of *E. coli* RecO with RecR (68). Although *E. coli* RecR does not interact with SSB or DNA (33,81), an SSB-Ct peptide allosterically stabilizes RecR_4_O complexes (68). Here we show that the SSB-Ct also affects the ssDNA binding activity of *E. coli* RecO and RecOR complexes.

## MATERIALS AND METHODS

### Buffers and reagents

Buffers were prepared with reagent grade chemicals using distilled, deionized water (Milli-Q system; Millipore Corp., Bedford, MA, USA). Spectrophotometric grade glycerol was from Alfa Aesar (Ward Hill, MA, USA). Buffer BTP is 20 mM Bis–Tris propane (pH 8.0 at 25°C, unless otherwise indicated), 50 mM NaCl unless otherwise indicated, 25% (v/v) glycerol, 1 mM TCEP. Tween-20 (0.002%) (Millipore Sigma, MO, USA) was added to Buffer BTP in the confocal microscope imaging experiments.

### Proteins, peptides and DNA

*Escherichia coli* RecO protein was overexpressed from plasmid pMCSG7 in *E. coli* strain BL21(DE3) pLysS (kindly provided by Dr Sergey Korolev, Saint Louis University) and purified using Ni-NTA affinity chromatography and a HiTrap Heparin HP affinity column (GE Healthcare, Chicago, IL, USA) after His-tag cleavage with TEV protease as described (36). The auto-inactivation-resistant S219V mutant of TEV protease with an N-terminal His-tag and C-terminal polyarginine tag (His-TEV(S219V)-Arg) was overexpressed from *E. coli* strain BL21(DE3) transformed with PRK793 and pRIL (Stratagene, San Diego, CA, USA) and purified as described (82). *E. coli* RecR protein was overexpressed from plasmid pMCSG7 in *E. coli* strain BL21 Rosetta 2(DE3)pLysS (kindly provided by Dr. Sergey Korolev) and purified using Ni-NTA affinity chromatography, followed by cleavage of His-tag with TEV protease as described (83). The concentrations of RecO and RecR in monomers were determined using extinction coefficients of ϵ_280_ = 2.44 × 10^4^ M^−1^cm^−1^ and ϵ_280_ = 5.96 × 10^3^ M^−1^cm^−1^, respectively, as determined from their amino acid sequences by SEDNTERP (84).

SSB-Ct peptide, composed of the 15 C-terminal amino acids (PSNEPPMDFDDDIPF) of *E. coli* SSB, was purchased from WatsonBio (Houston, TX, USA). The SSB-Ct peptide concentration was determined using an extinction coefficient of ϵ_258_ = 390 M^−1^ cm^−1^.

The ss oligodeoxynucleotides, 3’-Cy3-(dT)_L_, (dT)_L_ and (((dT)_3_ϵdA)*_m_*(dT)_3_ with *m* = 3, 8, 17) containing the fluorescent analogue, etheno(dA) (${\rm{\varepsilon }}$dA) (Glen Research, Sterling, VA, USA) (((dT)_3_ϵdA)*_m_*(dT)_3_ for *m* = 3, 8, 17) were synthesized and purified as described (9), and concentrations determined in units of nucleotides using the extinction coefficient and ϵ_260_ = *L*(8.1 × 10^3^) M^−1^ cm^−1^ for (dT)_L_, and ϵ_260_ = 1.1 × 10^5^, 2.6 × 10^5^ and 5.2 × 10^5^ M^−1^cm^−1^ for *m* = 3, 8, 17, respectively, for ((dT)_3_ϵdA)*_m_*(dT)_3_. Each strand of the double-stranded DNA (dsDNA) substrates were synthesized and purified as described (9). The sequences of the dsDNA are provided in the supplementary information. The concentration of each strand was determined using ϵ_260_ = 1.7 × 10^5^ M^−1^ cm^−1^, ϵ_260_ = 1.5 × 10^5^ M^−1^ cm^−1^, ϵ_260_ = 5.8 × 10^5^ M^−1^ cm^−1^ and ϵ_260_ = 5.7 × 10^5^ M^−1^ cm^−1^ for ds18A, ds18B, ds60A, and ds60B, respectively. The double stranded DNA was formed by annealing the two sets of complementary strands, ds18A and ds18B, and ds60A and ds60B, in equimolar amounts and then incubated in a water bath at 90°C for 5 min, then cooled slowly to 23°C.

### Analytical ultracentrifugation (AUC)

Sedimentation experiments were performed with an Optima XL-A analytical ultracentrifuge and An50Ti or An60Ti rotors (Beckman Coulter, Fullerton, CA, USA) at 25°C as described (18,68). Absorbance was monitored at 546 nm for the Cy3-labeled DNA (Figures 4–6, and S2a) and at 260 nm for unlabeled DNA (Figure S2b). Absorbance was also monitored at 230 nm. All sedimentation experiments were performed at least twice. In fact, each experiment was repeated using two entirely different RecO and RecR protein preps, yielding identical results. Furthermore, all of the sedimentation results are fully consistent with our previous studies of RecO and RecR interactions that were performed under the same solution conditions in the absence of DNA (68).

The densities and viscosities of the buffers at 25°C were determined using SEDNTERP (84). The partial specific volume, $\bar{\upsilon }$, of RecO and RecR were determined from independent sedimentation equilibrium experiments on each protein in buffer BTP (68). The values of $\bar{\upsilon }$ determined in buffer BTP are 0.734 ml/g for RecO and 0.711 ml/g for RecR. These values differ from the ones calculated using SEDNTERP by 1.2% and 2.7% for RecO and RecR, respectively (0.743 ml/g for RecO and 0.731 ml/g for RecR). The $\bar{\upsilon }$ of the SSB-Ct peptide was calculated using SEDNTERP, yielding 0.704 ml/g. $\bar{\upsilon }$ of 0.56 ml/g was used for DNA (85). In experiments involving more than one species, the partial specific volumes of complexes were calculated assuming additivity using Equation (1), where *n_i_* = number of moles of species ‘*i*’, *M_i_* = molecular weight of species ‘*i*’, and $\overline {{\upsilon }_i}$ = partial specific volume of each species ‘*i*’.


(1) $$\begin{equation*}\bar{\upsilon } = \frac{{\mathop \sum \nolimits_i {n}_i{M}_i\overline {{\upsilon }_i} }}{{\mathop \sum \nolimits_i {n}_i{M}_i}}\end{equation*}$$

#### Sedimentation velocity

Sedimentation velocity experiments were performed at 42,000 rpm with of 3’-Cy3-labeled (dT)_L_ (0.56 and 2.24 μM DNA molecules) and mixtures of RecO (2.24 μM), RecR (4.84, 8.96 and 17.9 μM), and SSB-Ct (13.4 μM). Sample (380 μl) and buffer (394 μl) were loaded into each sector of an Epon charcoal-filled two-sector centerpiece. Absorbance data were collected by scanning the sample cells at intervals of 0.003 cm and analyzed using Sedfit to obtain *c*(*s*) distributions (86). The *c*(*s*) distribution function defines the populations of species with different sedimentation rates and represents a variant of the distribution of Lamm equation solutions (86).

#### Sedimentation equilibrium

Sedimentation equilibrium experiments were analyzed to determine the molecular weight of the RecO(R)–DNA and complex species. Sedimentation equilibrium experiments were performed with 3’-Cy3-labeled (dT)_*L*_ (*L* = 15 or 35 nucleotides) (0.56 μM DNA molecules), RecO (2.24 μM), SSB-Ct (13.4 μM), and in the absence and presence of RecR (8.96 μM). Sample (110 μl) and buffer (120 μl) were loaded into each sector of an Epon charcoal-filled six-channel centerpieces. Absorbance data were collected by scanning the sample cells at intervals of 0.003 cm in the step mode with 5 averages per step. Samples were sedimented to equilibrium at the indicated rotor speeds (ranging from 18 000 to 28 000 rpm) starting with the lowest speed. The resulting absorbance profiles, *A_r_*, were analyzed by NLLS fitting to Eq. (2) as implemented in Sedphat (87) to obtain molecular weights using ‘Species Analysis with Mass Conservation Constraints’ model:


(2) $$\begin{eqnarray*}{A}_r &=& \mathop \sum \limits_{i = 1}^n {A}_{{r}_0,i} \cdot {\rm{exp}}\left[ {{M}_i\left( {1 - {{\bar{\upsilon }}}_i\rho } \right)\frac{{{\omega }^2}}{{2RT}}\left( {{r}^2 - r_0^2} \right)} \right] + {b}_r \end{eqnarray*}$$

where *r* is the distance from the center of rotation, *r_0_* is an arbitrary reference radius, ω is angular velocity, *T* is absolute temperature, *R* is the gas constant, *M_i_* is the molecular weight of species ‘*i*’, $\overline {{\upsilon }_i}$ = partial specific volume of each species ‘*i*’, ρ is the buffer density, ${A}_{{r}_0,i}$ is the absorbance of species ‘*i*’ at the reference position, and *b_r_* is a radial-dependent baseline offset. All sedimentation equilibrium experiments in this study were described by a single exponential and globally fit to a one species model (Figures 5 and 6C). When a two species model was attempted (nucleoprotein complex and unbound DNA), the fraction of the second species was less than 1%.

### Confocal microscopy

Confocal fluorescence measurements were performed using a Picoquant MT200 instrument (Picoquant, Germany). The microscope (Olympus IX-73, Japan) was equipped with a piezo scanner and a high numerical aperture water immersion objective (60 × 1.2 UPlanSApo Superapochromat, Olympus, Japan). Fluorophores were excited using a 485 nm pulsed laser (LDH PC-485, Picoquant, Germany) with a repetition rate of 20 MHz. Excitation power was monitored before the objective with a laser photodiode and optimized to avoid photobleaching and saturation of detectors to maintain a constant power for each set of measurements. Emitted photons were collected through the objective, passed through a dichroic mirror (ZT488/594rpc-UF3, Chroma, Bellows Falls, VT, USA), and filtered by a 100 μm pinhole (Thorlabs, Newton, NJ, USA). Photons were separated according to polarization using a polarizer beam splitter cube (Ealing, Scotts Valley, CA, USA) and further refined by a 642 ± 40 nm bandpass filter (E642/80m, Chroma, Bellows Falls, VT, USA) in front of the SPAD detectors (Excelitas, Waltham, MA, USA). Photons are counted and accumulated by a HydraHarp 400 TCSPC module (Picoquant, Germany) with 1 picosecond resolution (88).

Measurements were performed in uncoated polymer coverslip cuvettes (30 μl per well) (Ibidi, Germany), which significantly decrease the fraction of protein adhering to the surface compared to glass cuvettes. Measurements were performed at 23 ± 1°C in a temperature-controlled room, as detected on the microscope stage.

Imaging was performed using both XY and Z monodirectional scanning with 1 ms collecting steps with 256 × 256 pixels resolution. Excitation power for image collection was either 1.0 or 11 μW depending on sample concentration. Measurements were performed keeping a constant ratio between Cy3-labeled and unlabeled protein (labeled:unlabeled = 1:100) in buffer BTP with 0.002% Tween-20. Brightness thresholds were set at 50 and 1200 photons/pixel, which removed most of the background and prevented saturation in images. Images are colored in a hue scale running from blue at 100 photons/pixel to red at 800 photons/pixel.

### Light scattering

Light scattering at 90° was measured using a PTI QM-2000 fluorometer (Photon Technologies, Inc., Lawrenceville, NJ, USA) with excitation and emission wavelengths at 350 nm. ssDNA (1.9 ml of 25 nM DNA molecules) was titrated with RecO (5 μM stock) in a 3 ml quartz cuvette in buffer BTP. Samples were stirred throughout the experiments using magnetic stir bars. For experiments in the presence of SSB-Ct, SSB-Ct was pre-mixed with ssDNA at the start of the experiments. SSB-Ct (3.8 μM) was in 6-fold molar excess of the final concentration of RecO in the cuvette at the end of titration. The stock solution of RecO was also pre-mixed with 3.8 μM of SSB-Ct to keep [SSB-Ct] inside the cuvette constant throughout the titrations. Reference titrations were also performed in which protein titrant was added to a 1.9 ml of buffer that does not contain DNA both in the absence and presence of SSB-Ct. All sedimentation experiments were performed in duplicate, with each experiment using different RecO and RecR protein preps.

Light scattering intensities were normalized as in Eq. (3),


(3) $$\begin{equation*}{I}_{i,\ norm} = \frac{{{I}_i - {I}_0}}{{{I}_0}}\end{equation*}$$

where *I_i,norm_* is the normalized scattering intensity after ‘*i*’th injection of titrant (RecO), *I_i_* is the scattering intensity after ‘*i*’th injection of titrant, and *I*_0_ is the initial scattering intensity before injection of any titrants.

## RESULTS

### Large RecO–ssDNA aggregates form *in vitro* when RecO is in excess over DNA

Previous studies have shown that *E. coli* RecO can bind both ss and dsDNA and anneal complementary ssDNA when complexed with SSB (31,36). One of these studies (36) was carried out in buffer containing high concentrations of arginine (50 mM NaGlu and 50 mM Arg–HCl, pH 8.0) that suppresses RecO aggregation. The experiments reported here were performed in a more conventional buffer (buffer BTP (pH 8.0, 50 mM NaCl)) that tends to promotes RecO aggregation as discussed below.

**Figure 1.**
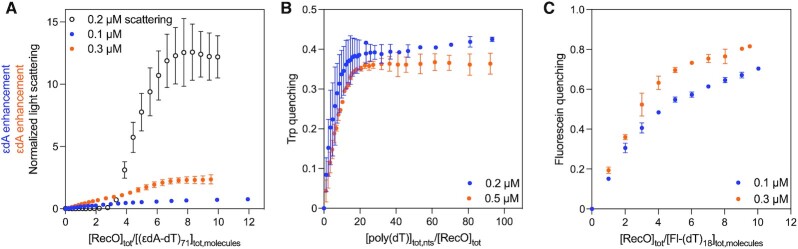
Fluorescence titrations show inconsistencies at different [RecO] and [ssDNA]. (**A**) Titration of 0.1 μM (blue) and 0.2 μM (orange) of (ϵdA-dT)_71_ with RecO while monitoring ϵdA enhancement. The two isotherms show different extents of enhancement. A plot of titration of 0.2 μM (ϵdA-dT)_71_ while monitoring light scattering is overlaid (empty circles). The onset of light scattering at ∼2.4 for [RecO]_tot_/[(ϵdA-dT)_71_]_tot, molecules_ correlates with the change in fluorescence titration, which suggests that inconsistencies in ϵdA enhancement may be due to light scattering. (**B**) Reverse titration of 0.2 μM (blue) and 0.5 μM (orange) RecO with poly(dT) while monitoring Trp quenching. (**C**) Titration of 0.1 μM (blue) and 0.3 μM (orange) fluorescein-labeled (dT)_18_ while monitoring fluorescein quenching. These data show different extents of quenching across multiple concentrations of titrants. These inconsistencies led us to suspect and investigate light scattering by RecO–DNA complexes.

We made several attempts to use fluorescence signals from either RecO or a labeled DNA to obtain quantitative information on the binding of RecO to ssDNA. These included titrations of RecO with ss oligodeoxythymidylates ((dT)_*L*_) monitoring RecO Trp fluorescence quenching and fluorescein fluorescence quenching or anisotropy using 5’-fluorescein labeled (dT)_L_ (Fl-(dT)_*L*_). Finally, we used ssDNA composed of dT interspersed with the fluorescent analogue, etheno(dA) (${\rm{\varepsilon }}$dA) (((dT)_3_${\rm{\varepsilon }}$dA)*_m_*(dT)_3_ with *m* = 3, 8, 17) and monitored the enhancement of ${\rm{\varepsilon }}$dA fluorescence upon RecO binding. However, none of these titrations showed consistent results when performed at multiple RecO or DNA concentrations as shown in Figure 1. Titrations of a 71-nucleotide long ssDNA containing ${\rm{\varepsilon }}$dA, (((dT)_3_${\rm{\varepsilon }}$dA)_17_(dT)_3_, referred to as (${\rm{\varepsilon }}$dA-dT)_71_), with RecO at two DNA concentrations (0.1 and 0.2 μM) in buffer BTP (pH 8.0, 50 mM NaCl) at 25°C show different plateau levels for the normalized enhancement of ${\rm{\varepsilon }}$dA fluorescence at saturating RecO (Figure 1A, filled circles). Titrations of (${\rm{\varepsilon }}$dA-dT)_35_ with RecO yielding similar inconsistencies (Figure S1). Reverse titrations of RecO with poly(dT) monitoring Trp fluorescence quenching (Figure 1B) also show inconsistent maximum quenching values. Titrations of fluorescein labeled ssDNA, Fl-(dT)_18_, with RecO showed similar inconsistencies (Figure 1C). These results suggest that large complexes form during the titrations and that the resulting light scattering interferes with the spectroscopic experiments.

To examine this further, we monitored light scattering during a titration of 0.2 μM (${\rm{\varepsilon }}$dA-dT)_71_ with RecO under the same solution conditions (buffer BTP, pH 8.0, 50 mM NaCl, 25°C) used to monitor ${\rm{\varepsilon }}$dA fluorescence as described in Methods. Figure 1A (empty circles) shows the absence of light scattering at low RecO/DNA ratios, however significant light scattering appears at RecO/DNA ratios > 3. The onset of light scattering at high RecO/DNA ratios explains the inconsistent results of the fluorescence titrations in Figure 1. Furthermore, the significant light scattering suggests that large RecO–ssDNA complexes form when RecO is in excess over DNA, resulting either from aggregation, phase separation (89), or both. This led us to further investigate the formation of these large RecO–ssDNA complexes by monitoring light scattering.

**Figure 2.**
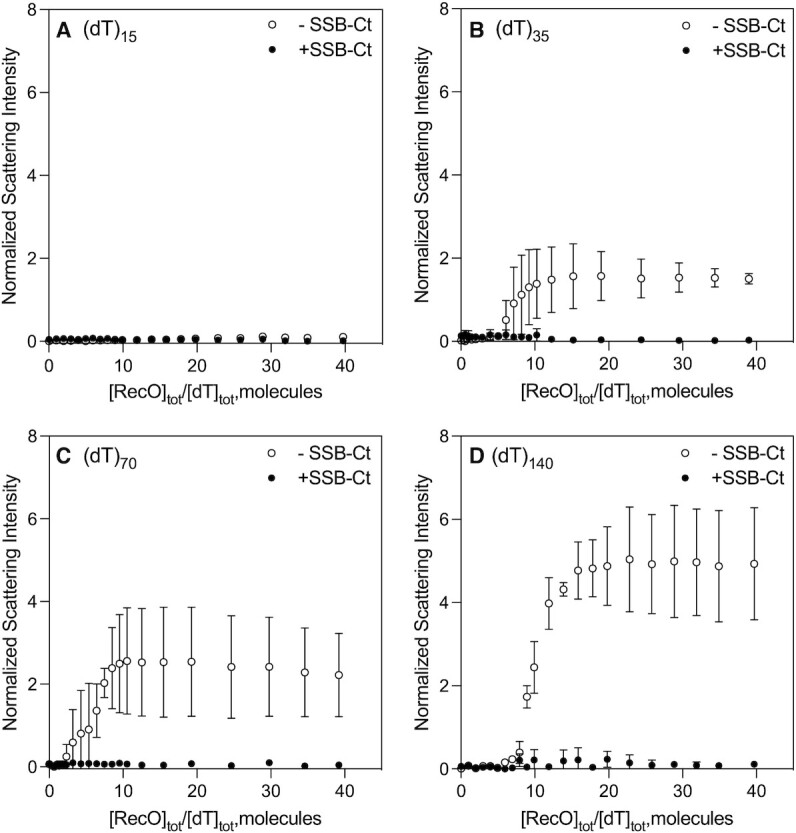
SSB-Ct prevents light scattering by RecO–ssDNA complexes. Titrations of ssDNA (25 nM) with RecO (5 μM stock) while monitoring light scattering in the absence (empty circles) and presence (filled circles) of SSB-Ct for (**A**) (dT)_15_, (**B**) (dT)_35_, (**C**) (dT)_70_ and (**D**) (dT)_140_. The ordinate of each panel shows normalized light scattering intensity as described in Materials and Methods (Eq. 3). In the presence of SSB-Ct, no significant light scattering is observed except for a small increase in light scattering intensity for (dT)_140_ (panel (d), filled circles). In the absence of SSB-Ct, however, light scattering is observed at RecO to (dT)_*L*_ molar ratio of 5.2, 6 and 7.1 for (dT)_35_, (dT)_70_ and (dT)_140_, respectively. The increase in the molar ratio until the onset of scattering with increasing ssDNA length suggests that a critical binding density must be reached for aggregation.

We performed titrations of a series of oligodeoxythymidylates, (dT)_*L*_ (25 nM DNA molecules), of different lengths (*L* = 15, 35, 70 and 140 nucleotides) with RecO (5 μM stock) monitoring light scattering in buffer BTP (pH 8.0, 50 mM NaCl at 25°C). Scattering intensities were normalized using Eq. (3) as described in Methods. Reference titrations of RecO into buffer showed no scattering, and no light scattering was observed upon titrating (dT)_15_ with RecO (Figure 2A, open circles). This indicates either the absence of (dT)_15_ binding to RecO or formation of complexes that do not result in aggregation or phase separation. However, significant light scattering was observed for RecO titrations of (dT)_35_, (dT)_70_ and (dT)_140_. Furthermore, the RecO to (dT)_*L*_ ratio at which the onset of light scattering occurs increases with increasing DNA length at RecO to (dT)_*L*_ molar ratios of 5.2 for (dT)_35_, 6 for (dT)_70_ and 7.1 for (dT)_140_ (Figure 2B–D). This suggests that a critical binding density of RecO on the ssDNA is required to initiate light scattering. The maximum scattering intensities also increase with increasing DNA length (Figure 2, empty circles). The observation of higher scattering intensities with longer (dT)_L_ suggests that multiple RecO molecules binding to a longer DNA molecule facilitate formation of large RecO–ssDNA complexes, and that the size of the complexes increases with increasing DNA length.

Similar scattering experiments were performed with dsDNA of 18 and 60 bp (ds18 and ds60, respectively). Figure S2 shows increases in scattering intensities for both ds18 and ds60 with the onset of scattering occurring at higher RecO/DNA ratios for the longer DNA as in the case of ssDNA. However, a notable difference is that scattering is readily observed even for the short 18 bp DNA whereas ssDNA of similar length, (dT)_15_, did not exhibit observable scattering. This indicates that RecO binding to dsDNA is more prone to aggregation compared to ssDNA of similar length.

**Figure 3.**
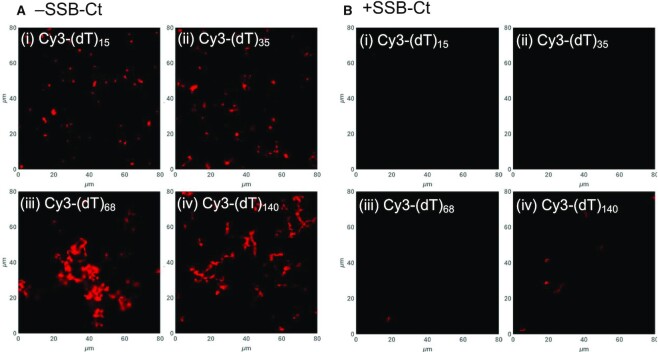
Amorphous RecO-(dT)_L_ aggregates form irreversibly with structures increasing in size for longer (dT)_*L*_. Images of RecO-(dT)_*L*_ obtained by confocal microscopy in the (**A**) absence and in the (**B**) presence of SSB-Ct peptide (24 μM) for (i) (dT)_15_, (ii) (dT)_35_, (iii) (dT)_68_ and (iv) (dT)_140_ from mixtures of ssDNA (200 nM DNA molecules) and 20-fold molar excess of RecO (4 μM) where light scattering intensity values have at least reached the maximum values for each (dT)_*L*_ as shown in Figure 2. Stock (dT)_*L*_ solutions were prepared for final molar ratio of 3’-Cy3-labeled to unlabeled DNA as 1:100. The Cy3-labeled and unlabeled counterparts were the same length except for Cy3-(dT)_68_ and unlabeled (dT)_70_. In the absence of SSB-Ct, amorphous aggregates of RecO-(dT)_*L*_ complexes were observed with increase in size for longer (dT)_*L*_. In the presence of 6-fold SSB-Ct (24 μM) over RecO, however, RecO-(dT)_*L*_ aggregates are invisible for (dT)_15_ and (dT)_35_, and only a few small structures are visible for (dT)_68_ and (dT)_140_.

We next used fluorescence confocal microscopy to examine the RecO-(dT)_*L*_ complexes formed in the presence of excess RecO that result in light scattering. Experiments were performed using a 20-fold molar excess of RecO (4 μM RecO) over ssDNA (200 nM) where light scattering intensities reached a plateau for all (dT)_*L*_ with *L* = 35, 70 and 140 nts (Figure 2). The DNA used contained a mixture of unlabeled (dT)_*L*_ (200 nM DNA molecules) and a small amount of Cy3-labeled (dT)_*L*_ (1:100 molar ratio) (containing a single Cy3 covalently attached to the 3’-end of the DNA) of the same length except for the experiments with (dT)_70_, which were performed by mixing with 3’-Cy3-(dT)_68_. As shown in Figure 3A, we observe amorphous fluorescent structures under these conditions for all (dT)_L_. These structures did not merge or split over several minutes of imaging, suggesting that the structures are not dynamic, but are solid aggregates. These aggregates appeared larger and more elongated for the longer (dT)_L_, consistent with the higher light scattering intensity for these longer DNA lengths (Figure 2). Interestingly, Figure 3Ai shows that aggregates form even for RecO binding to (dT)_15_, although significant light scattering was not observed at this RecO/(dT)_15_ ratio (Figure 2A). It is possible that the confocal imaging is more sensitive in detecting small aggregates than is light scattering.

### SSB-ct peptide binding to RecO inhibits large RecO–ssDNA complex formation

*Escherichia coli* single stranded binding (SSB) protein interacts with RecO via the last nine amino acids of SSB’s C-terminal intrinsically disordered tails (SSB-Ct) (26,31,33,36,57). We therefore examined whether the interaction of RecO with DNA is influenced by its binding to the SSB-Ct. For these studies we used a 15 amino acid peptide (PSNEPPMDFDDDIPF), that contains the last 15 amino acids of the SSB-Ct, including the region that binds RecO. Our previous studies showed that SSB-Ct forms a 1:1 complex with RecO with equilibrium constant *K* = (1.2 ± 0.3) × 10^7^ M^−1^ in buffer BTP (pH 8.0, 50 mM NaCl, 25°C) (57). Based on this binding affinity, a 6-fold molar excess of SSB-Ct (3.8 μM) over RecO will result in >97% saturation of RecO at 0.63 μM of RecO. We therefore performed all of the following light scattering experiments with 3.8 μM of SSB-Ct that was pre-mixed with (dT)_*L*_, such that SSB-Ct was in a 6-fold molar excess over the final RecO concentration. The RecO solution also contained SSB-Ct at 3.8 μM in order to maintain a constant concentration of SSB-Ct during the titration. When RecO was mixed with (dT)_*L*_ (*L* = 15, 35 and 70 nucleotides) (Figure 2, filled circles) in the presence of SSB-Ct, no significant light scattering was observed. However, a slight increase in scattering intensity was still observed for the longer (dT)_140_ (Figure 2D) with the maximum scattering intensity ∼6-fold lower than in the absence of SSB-Ct.

Experiments with RecO and dsDNA in the presence of SSB-Ct exhibited an increase in scattering intensity for both ds18 and ds60 in contrast to ssDNA (Figure S2). In the presence of SSB-Ct, however, the onset of scattering occurred at higher RecO/DNA ratios than in the absence of SSB-Ct (0.5 and 7 for ds18, and 2.5 and 18 for ds60, respectively). The maximum scattering intensities were similar for ds18 in the absence and in the presence of SSB-Ct, but the maximum intensity was reduced ∼3-fold for ds60 in the presence of SSB-Ct.

We next used confocal fluorescence microscopy to examine the effect of the SSB-Ct peptide (24 μM) on mixtures of RecO and(dT)_*L*_ at a 20-fold molar excess of RecO (4 μM) over (dT)_*L*_ (200 nM DNA molecules, labeled:unlabeled = 1:100 molar ratio) as before. Images showed mostly black background indicating that the binding of SSB-Ct to RecO significantly reduced the formation of the aggregated RecO–DNA structures (Figure 3B). No aggregates were observed for the RecO-(dT)_15_ and (dT)_35_ complexes, and only a few small fluorescent aggregates were observed for the RecO-(dT)_68_ and (dT)_140_ complexes, significantly reduced in size and number (Figure 3Biii, iv). This is consistent with the significantly reduced light scattering intensities observed in the presence of SSB-Ct (Figure 2, filled circles). Hence, the binding of SSB-Ct to RecO reduced its tendency to form aggregates with ssDNA. However, aggregates formed in the absence of SSB-Ct did not dissolve upon addition of SSB-Ct. It was unclear from these results whether the decreased aggregation was due to a lower binding affinity of SSB-Ct-bound RecO to (dT)_*L*_ or to a difference in the properties of a SSB-Ct-RecO-(dT)_*L*_ ternary complex. To clarify this, we performed sedimentation velocity experiments as described below.

### SSB-ct peptide enhances RecO affinity for ssDNA

**Figure 4.**
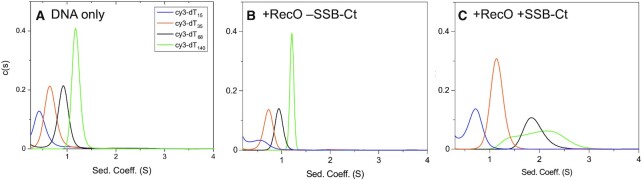
SSB-Ct peptides enhance RecO binding to ssDNA. (**A**) Sedimentation velocity *c*(*s*) distribution profiles (monitored at 546 nm) of Cy3-labeled (dT)_*L*_ (0.56 μM) show single symmetric peaks with weight average sedimentation coefficients 0.5 S, 0.7 S, 0.9 S and 1.2 S, respectively, for (dT)_15_ (blue), (dT)_35_ (orange), (dT)_68_ (black), and (dT)_140_ (green). (**B**) A 4-fold molar excess of RecO (2.24 μM) is added to Cy3-labeled (dT)_*L*_ (0.56 μM). The *c*(*s*) distribution profiles show weight average sedimentation coefficients of 0.6 S, 0.7 S, 1.0 S and 1.2 S, respectively, for (dT)_15_, (dT)_35_, (dT)_68_ and (dT)_140_. (**C**) A 6-fold molar excess of SSB-Ct (13.4 μM) over RecO is added to RecO (2.24 μM) and (dT)_*L*_ (0.56 μM). The species distributions show weight average sedimentation coefficients of 0.7 S, 1.2 S, 1.9 S and 2.3 S, respectively.

We used analytical ultracentrifugation to examine the binding of RecO to ssDNA labeled with a Cy3 probe on its 3’ end (Cy3-(dT)_*L*_). For these experiments, we used only a 4-fold molar excess of RecO over Cy3-(dT)_*L*_, a molar ratio such that no light scattering is observed for any of the (dT)_*L*_ examined. Experiments were performed by monitoring absorbance at 546 nm, which only detects the Cy3-(dT)_L_ and Cy3-(dT)_L_-bound to protein. Sedimentation velocity experiments were performed with 0.56 μM Cy3-(dT)_*L*_ and 2.24 μM RecO in the absence and presence of SSB-Ct. Figure 4A shows the *c*(*s*) distributions for Cy3-(dT)_*L*_ (*L* = 15, 35, 68 and 140) in the absence of RecO. A single symmetric *c*(*s*) peak is observed for each DNA, indicating that each is a single homogeneous species. Weight average sedimentation coefficients of 0.5 S, 0.7 S, 0.9 S and 1.2 S were estimated for Cy3-(dT)_15,_ Cy3-(dT)_35,_ Cy3-(dT)_68_ and Cy3-(dT)_140_, respectively. Upon addition of a four-fold molar excess of RecO over ssDNA in the absence of SSB-Ct, the *c*(*s*) species distributions still showed only single peaks with only slight increases in sedimentation coefficients of 0.6 S, 0.7 S, 1.0 S and 1.2 S, indicating weak binding of RecO to ssDNA under these conditions (Figure 4B).

However, when a 6-fold molar excess of SSB-Ct (13.4 μM) was included with RecO (2.24 ${\rm{\mu }}$M), we observed increases in the weight average sedimentation coefficients of 0.7 S, 1.2 S, 1.9 S and 2.1 S for Cy3-(dT)_15,_ Cy3-(dT)_35,_ Cy3-(dT)_68_ and Cy3-(dT)_140_, respectively (Figure 4C), indicating increased RecO binding to (dT)_*L*_. Hence, SSB-Ct binding to RecO enhances the RecO-(dT)_*L*_ binding affinity. We also note that the *c*(*s*) species distributions for Cy3-(dT)_68_ and Cy3-(dT)_140_ in Figure 4C are noticeably asymmetric indicating that multiple RecO-(dT)_L_ complexes form when SSB-Ct-RecO binds to these longer ssDNA molecules.

### One RecO molecule binds to (dT)_15_ while two RecO molecules can bind to (dT)_35_ in the presence of SSB-ct

**Figure 5.**
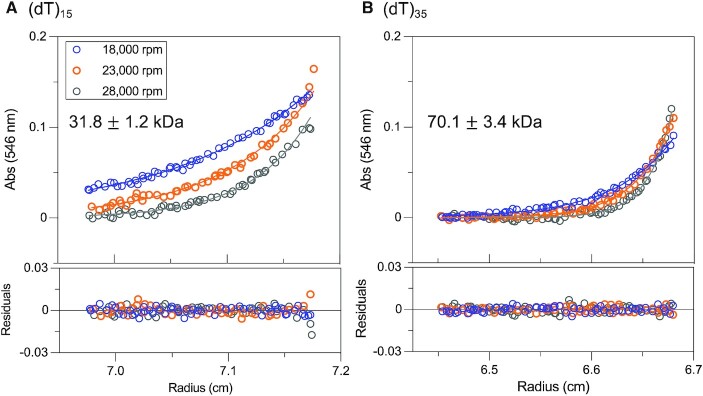
At least one SSB-Ct-bound RecO binds to (dT)_15_ and two SSB-Ct-bound RecO bind to (dT)_35_. Sedimentation equilibrium experiments were performed for RecO (2.24 μM), SSB-Ct (13.4 μM), and Cy3-(dT)_*L*_ (*L* = 15 (**A**), 35 (**B**)) (0.56 μM) at three rotor speeds (18 000 (blue), 23 000 (orange) and 28 000 (gray) rpm) and monitored at 546 nm. The sedimentation profiles were described by single exponentials and were globally fitted to a one-species model with mass constraint to yield estimated MW of RecO-(dT)_*L*_ complexes as 31.8 ± 1.2 kDa, consistent with one SSB-Ct-bound RecO forming a complex with (dT)_15_ (expected MW 33.8 kDa), and 70.1 ± 3.4 kDa, consistent with two molecules of SSB-Ct-bound RecO forming a complex with (dT)_35_ (expected MW 69.5 kDa). This shows that at least one RecO can bind (dT)_15_ and two RecO can bind (dT)_35_ in the presence of SSB-Ct.

The N-terminal domain of RecO contains the DNA binding domain (83), however, there is no information available on the occluded site size (90) or the ssDNA contact size for RecO binding to ssDNA. This information is important since these properties constrain the RecO binding stoichiometries for each (dT)_L_. In order to assess these stoichiometries, we performed sedimentation equilibrium experiments using a four-fold molar excess of RecO (2.24 μM) over Cy3-(dT)_15_ or Cy3-(dT)_35_ (0.56 μM), and a six-fold molar excess of SSB-Ct (13.4 μM) over RecO at three rotor speeds (18 000, 23 000 and 28 000 rpm). The sedimentation equilibrium profiles showed only a single exponential for the RecO complexes with both Cy3-(dT)_15_ and Cy3-(dT)_35_. This is consistent with the *c*(*s*) species distributions from sedimentation velocity that showed only a single species under these same conditions (Figure 4). The sedimentation equilibrium data were therefore fit to a one species model with mass constraint (Eq. 2) to obtain molecular weights of 31.8 ± 1.2 kDa for the SSB-Ct-RecO-(dT)_15_ complex (Figure 5A) and 70.1 ± 3.4 kDa for the SSB-Ct-RecO-(dT)_35_ complex (Figure 5B). These are consistent with the expected molecular weights of 33.8 kDa and 69.5 kDa, respectively, for a complex containing one SSB-Ct-RecO bound to (dT)_15_ and two SSB-Ct-RecO bound to (dT)_35_.

### RecR inhibits RecO-(dT)_L_ aggregation

We have shown that RecR exists in a dimer-tetramer equilibrium under the conditions of our experiments and that RecO binding promotes RecR tetramer formation (buffer BTP, pH 8.0, 50 mM NaCl, 25.0°C) (68). Furthermore, up to two RecO molecules can bind to a RecR tetramer. Under our solution conditions, a 4-fold molar excess of RecR over RecO (monomer units) yields a mixture of RecR_4_O and RecR_4_O_2_, but primarily RecR_4_O_2_ and some excess RecR dimer (68). However, in the presence of SSB-Ct, RecR_4_O is favored and becomes the primary species. As *E. coli* RecR does not interact with DNA (81,91), the following experiments were performed with a four-fold molar excess of RecR (8.96 μM) over RecO (2.24 μM) in order to avoid free RecO protein in the mixture. Surprisingly, we did not observe aggregation under these conditions, even in the absence of SSB-Ct for any length of (dT)_*L*_ (Figure S3a). This contrasts with significant aggregation in the absence of RecR, particularly for longer DNA (Figure 3A). In the presence of RecR, aggregates were also not observed in the presence of SSB-Ct (Figure S3b). This demonstrates a second allosteric effect, by RecR, on binding of RecO to ssDNA. However, it is unclear whether the presence of RecR abolished binding of RecO to ssDNA or changed the properties of the RecOR-(dT)_L_ complex compared to RecO-(dT)_L_ resulting in the inhibition of aggregation. To clarify this, we performed sedimentation velocity experiments with RecOR-ssDNA in the absence and presence of SSB-Ct.

### Effect of SSB-ct on ssDNA binding to RecOR complexes

**Figure 6.**
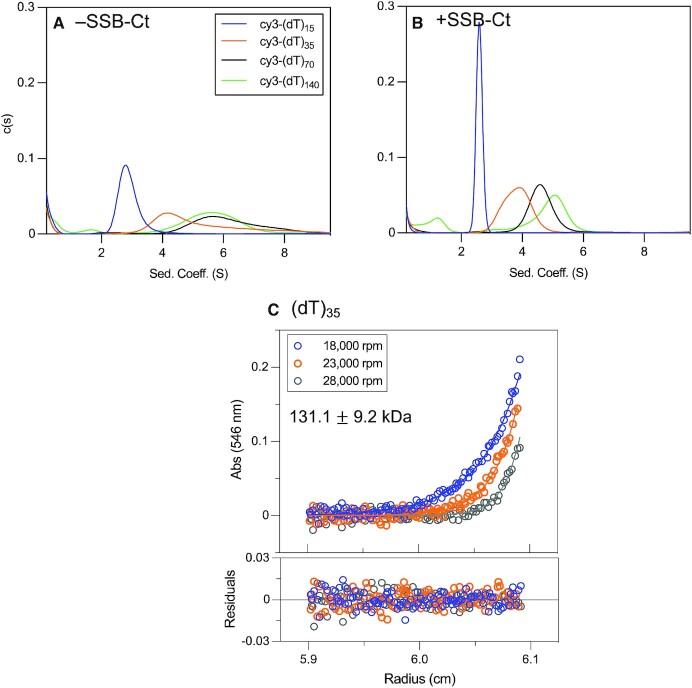
SSB-Ct-RecR_4_O complexes binds to ssDNA. Sedimentation velocity distribution (c(s)) profiles for Cy3-labeled (dT)_*L*_ (0.56 μM), RecO (2.24 μM) and RecR (8.96 μM) (monitored at 546 nm) in the (**A**) absence and (**B**) presence of SSB-Ct (13.4 μM) for (dT)_15_ (blue), (dT)_35_ (orange), (dT)_68_ (black) and (dT)_140_ (green). In the absence of SSB-Ct, distribution profiles show weight average sedimentation coefficients of 2.9 S, 5.4 S, 6.3 S and 5.7 S, for the four respective lengths of ssDNA. (**B**) In the presence of SSB-Ct, the weight average sedimentation coefficients have decreased to 2.6 S, 3.8 S, 4.7 S and 4.8 S. (**C**) Sedimentation equilibrium experiments were performed at three rotor speeds (18 000 (blue), 23 000 (orange) and 28 000 (gray) rpm) for Cy3-(dT)_15_ (0.56 μM DNA molecules), RecO (2.24 μM), RecR (8.96 μM) and SSB-Ct (13.4 μM) and monitored at 546 nm. The data were described by a single exponential and fitted to a one-species model with mass constraint to yield a MW estimate of 131.1 ± 9.2 kDa, which represents SSB-Ct-bound RecR_4_O binding to one (dT)_15_ (expected MW 121.5 kDa).

While *E. coli* RecR does not bind to either SSB-Ct or DNA (33,81), we have shown that SSB-Ct binding to RecO shifts the RecR_4_O/RecR_4_O_2_ equilibrium to favor a RecR_4_O complex (68). In order to study the effect of SSB-Ct on ssDNA binding to the RecOR complexes, we performed sedimentation velocity experiments at a 1:4 molar ratio of [RecO]:[RecR] (2.24 μM RecO and 8.96 μM RecR) with Cy3-labeled (dT)_*L*_ (0.56 μM) (*L* = 15, 35, 68 and 140 nucleotides) in the absence and presence of SSB-Ct (13.4 μM), monitoring Cy3 absorbance. This [RecO]:[RecR] molar ratio yields primarily RecR_4_O_2_ in the absence of SSB-Ct and RecR_4_O in the presence of SSB-Ct (68). The weight average sedimentation coefficients of each Cy3-(dT)_*L*_ (*L* = 15, 35, 68, 140) alone are 0.5 S, 0.7 S, 0.9 S and 1.2 S (Figure 4A), but these increase significantly to 2.9 S, 5.4 S, 6.3 S and 5.7 S in the presence of a four-fold excess of RecR over RecO (Figure 6A), indicating that RecOR complexes bind to all (dT)_L_ in the absence of SSB-Ct. This should be compared to the very small changes in sedimentation coefficients upon RecO binding in the absence of RecR (Figure 4B). In the presence of RecR, we also note that the species distributions are wide and asymmetric, indicating the presence of multiple bound species (Figure 6A).

Adding SSB-Ct (13.4 μM) to the RecR (8.96 μM), RecO (2.24 μM) and (dT)_*L*_ (0.56 μM) mixture results in decreases in the weight average sedimentation coefficients to 2.6 S, 3.8 S, 4.7 S and 4.8 S (Figure 6B). This could reflect either dissociation of RecO from RecR_4_O_2_ to form RecR_4_O in the presence of SSB-Ct (68), a destabilization of RecOR-(dT)_L_ complexes, or both. For the longer (dT)_*L*_ (*L* = 35, 68, 140), we observe wide, asymmetric *c*(*s*) distributions at >3 S indicating that multiple SSB-Ct-RecOR-(dT)_*L*_ complex species are present. In addition to the RecOR-(dT)_*L*_ species, experiments with (dT)_140_ (Figure 6, green) show a small peak at ∼1.3 S both in the absence and presence of SSB-Ct with an increase in the peak area in the presence of SSB-Ct (6.3% in the absence of SSB-Ct, 20.4% in the presence of SSB-Ct). The sedimentation coefficient of this peak (1.3 S) may represent either unbound (dT)_140_ or a RecO-(dT)_140_ complex. Since the presence of multiple species complicates the identification of the RecOR-ssDNA complexes for the longer (dT)_*L*_, we performed sedimentation equilibrium experiments on the mixture of RecO, RecR, SSB-Ct and Cy3-(dT)_15_, which displays a symmetric sedimentation velocity peak at 2.9 S, suggesting a homogeneous species (Figure 6B). Furthermore, we note that the shape of the peak for the RecO-(dT)_15_ species changes (Figure 6A and B, blue) from a wider asymmetric distribution in the absence of SSB-Ct to a single symmetric peak in the presence of SSB-Ct.

Sedimentation equilibrium experiments were performed to estimate the MW and thus identify the composition of the RecOR complexes. A mixture of RecO (2.24 μM) RecR (8.96 μM) and Cy3-(dT)_15_ (0.56 μM) and SSB-Ct (13.4 μM) was examined (Figure 6C). Each sedimentation equilibrium profile can be described by a single exponential, consistent with the single symmetric c(s) peak observed for the SSB-Ct-RecOR-(dT)_15_ complex by sedimentation velocity (Figure 6B, blue). Therefore, the sedimentation equilibrium data were fit to a one species model with mass constraint (Eq. 2), yielding an estimated MW of 131.1 ± 9.2 kDa, consistent, within error, with a RecR_4_O-SSB-Ct complex bound to one (dT)_15_ (121.2 kDa). Even the upper limit of the MW estimate is 10.3 kDa less than the predicted MW (155.6 kDa) for two (dT)_15_ molecules bound to RecR_4_O_2_ along with two SSB-Ct molecules. In contrast to the symmetric peak observed in the presence of SSB-Ct, the asymmetric peak for (dT)_15_ in in the absence of SSB-Ct (Figure 6A (blue)) may reflect a mixture of RecR_4_O-(dT)_15_ and RecR_4_O_2_-(dT)_15_ complexes. Therefore, the transition from RecR_4_O_2_ to RecR_4_O upon binding of SSB-Ct is observed even when the RecOR complexes are bound to (dT)_15_.

## DISCUSSION

### Allosteric effects of SSB-ct and RecR on RecO–ssDNA aggregation

We showed previously that the SSB-Ct exerts an allosteric effect on RecOR complex formation (68). Two types of RecOR complexes can be formed: RecR_4_O and RecR_4_O_2_, and SSB-Ct binding to RecO preferentially stabilizes the RecR_4_O complex. In this study we report evidence for a second allosteric effect of the SSB-Ct on RecO binding to DNA. Aggregation of RecO–ssDNA complexes is inhibited when SSB-Ct is pre-bound to RecO. This is not due to the inability of SSB-Ct-bound RecO to interact with ssDNA, as we have shown that (dT)_L_ from 15 to 140 nts interacts with SSB-Ct-bound RecO when RecO is in excess over ssDNA. We also observe that RecO–ssDNA aggregation is completely inhibited in the presence of RecR even in the absence of SSB-Ct, demonstrating an allosteric effect of RecR on binding of RecO to ssDNA.

Using a pull-down assay with (dT)_45_ and (dT)_70_, Ryzhikov et al. (36) showed that a complex of RecO–RecR–SSB–ssDNA can form with full-length SSB protein, as RecO and RecOR interact with both free and SSB-bound DNA (36). These interactions were examined in quite different solution conditions (50 mM NaGlu and 50 mM Arg–HCl, pH 8.0) that inhibit RecO aggregation (36).

**Figure 7.**
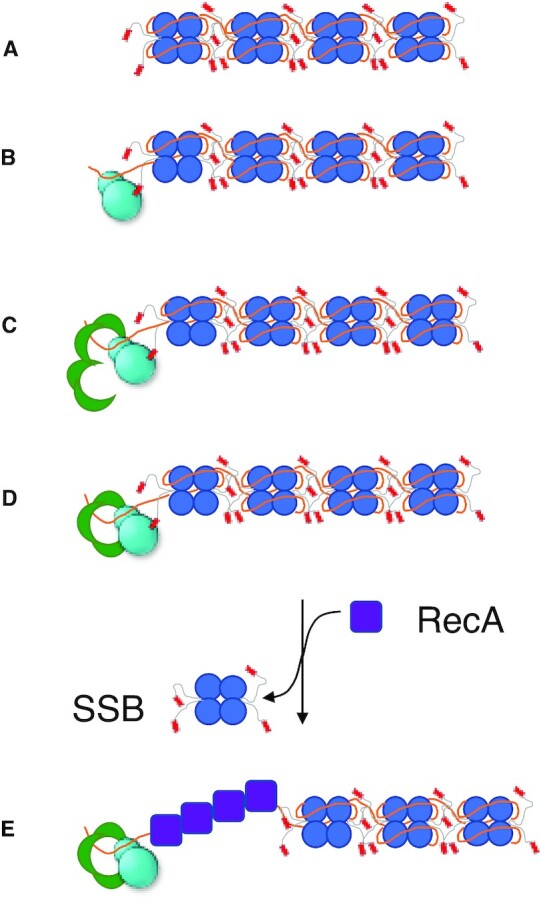
A cartoon model of RecO, RecR, SSB and ssDNA assembly. The DNA binding domains of SSB tetramer are represented by blue circles, the IDLs in gray lines, and the tip regions in red rectangles. RecO is represented in cyan with a smaller sphere representing the N-terminal DNA binding domain and the larger sphere representing the C-terminal domain. RecR tetramer is shown in four green arcs forming a ring. ssDNA is shown in orange. RecA is shown in purple squares. (**A**) SSB is tightly bound to ssDNA in the (SSB)_65_ binding mode. (**B**) ssDNA-bound SSB recruits RecO via the acidic tip region. Binding to RecO induces a binding mode change in SSB to (SSB)_35_, which occupies two subunits of SSB on average, releasing ssDNA, and RecO can bind to this region with enhanced affinity. (**C**) Tetramerization is promoted for RecR, in a dimer-tetramer equilibrium, when binding to RecO. (**D**) When RecO is bound to SSB-Ct, a RecR_4_O complex formation is stabilized over RecR_4_O_2_, possibly making more SSB-Ct available for following steps. (**E**) The ssDNA release due to a binding mode change facilitate dissociation of SSB from ssDNA and loading of RecA to the free ssDNA region.

The potential biological significance of the RecO–ssDNA aggregates is not clear. Although prior binding to SSB-Ct inhibits the aggregation process, the irreversible nature of the RecO–ssDNA aggregates, even upon addition of SSB-Ct, suggests that the aggregates may not interact with SSB-Ct. As the RecOR pathway for loading RecA requires a direct interaction between RecO and SSB (77), it is likely that RecOR and DNA interact under conditions where aggregates do not form. In fact, it has been suggested that RecO first interacts with SSB-Ct (36,68), which promotes RecO binding to DNA while remaining bound to SSB-Ct (Figure 7). In this sequence of events, it is unlikely that aggregates would form. Furthermore, subsequent formation of the RecOR complex also inhibits aggregation. While the RecO concentration has not been determined accurately, the relative abundance of RecO, RecR and SSB can be inferred from the reported rates of protein synthesis in E. coli. Li, et al. (92), reported that the rate of SSB synthesis is much faster than that of RecR, and RecR synthesis is faster than RecO synthesis in MOPS complete media growth conditions as follows: SSB (14444 molecules per generation) >> RecR (1342 molecules per generation) > RecO (85 molecules per generation) (92). At such ratios where RecR is in large excess over RecO, the RecR_4_O complex should be populated rather than RecR_4_O_2_ complexes (68). Together with the observation that the formation of RecO–DNA aggregates are inhibited by RecR(Figure S3), we suggest that the functional state of the quaternary SSB–RecO–RecR–DNA complex to be soluble without any RecO–DNA aggregates.

Harami *et al.* (93) have reported condensate formation of SSB and that RecQ, another SIP, can bind to SSB within these condensates that may function to store SSB that can be released rapidly upon DNA damage or stress. SSB condensate formation is promoted by potassium glutamate, the major monovalent *E. coli* salt (21). A translesion synthesis polymerase Pol IV has also been proposed to function by forming a pool of SSB and Pol IV at the site of DNA replication stress. It is possible that RecO or a RecOR complex can also form a condensate together with SSB and other SIPs to play a similar role as RecQ and Pol IV.

Under certain solution conditions requiring the presence of acetate or glutamate salts, SSB protein can promote the condensation or collapse of polymeric ssDNA beyond the compaction that occurs due to wrapping of ssDNA around the SSB tetramer in the (SSB)_65_ complex, indicating long-range, non-nearest neighbor intramolecular interactions (18–21). The binding of RecO results in a further condensation of the ssDNA-SSB nucleoprotein complex, possibly by inducing a change in the binding mode of SSB (20). Such a change in the binding mode of SSB due to SSB-RecO interaction was also suggested by Ryzhikov *et al.* (36), which would result in a release of ssDNA, rendering the nucleotides available for RecO to bind and bridge distant sites on the DNA. Furthermore, it has been suggested that RecO(R) can interact with long ssDNA–SSB filaments in trans to facilitate annealing of complementary strands by RecO (20). Our observation of reduced aggregation of RecO–ssDNA in the presence of SSB-Ct peptides, without the DNA binding domains of SSB, suggests that there is also a change in the properties of RecO–ssDNA complexes, such that the complex remains soluble during annealing even at or beyond the critical binding density that promotes aggregation. This additional condensation of SSB-ssDNA complex in the presence of RecO and RecR brings remote regions of ssDNA together, which may facilitate a homology search by RecA (94).

### SSB-ct affects both RecO and RecOR binding to ssDNA

In addition to the allosteric effect of SSB-Ct that inhibits RecO–ssDNA aggregation, we also observed that SSB-Ct affects binding of both RecO and RecOR to ssDNA. Since the SSB-Ct interacts only with RecO in a hydrophobic pocket remote from the N-terminal DNA binding domain and not with RecR or ssDNA (33,81,91), we suggest that the effects of SSB-Ct on the DNA binding activity of RecO are allosteric.

Ryzhikov *et al.* (36) have shown that ssDNA bound to SSBΔC8, a construct which lacks the C-terminal 8 amino acids of the acidic tip that binds RecO, does not bind to RecOR as well as ssDNA-bound to wild-type SSB, suggesting that the SSB-Ct facilitates recruitment of RecOR to ssDNA (36). Our observation of enhanced binding of RecO–SSB-Ct to ssDNA would ensure that RecO remains bound to ssDNA until a RecOR complex is formed. Since RecR exists in a dimer-tetramer equilibrium and that RecO facilitates RecR tetramer formation (68), it is possible that the RecR tetramer is loaded by the SSB–RecO complex as a ring around ssDNA. Furthermore, the binding of the SSB-Ct to RecOR favors the RecR_4_O species, rather than RecR_4_O_2_ (68). Based on this, we hypothesize that the SSB-RecR_4_O-ssDNA is the functional complex involved in RecA loading.

Formation of the SSB–RecR_4_O–ssDNA complex may induce a change in ssDNA binding mode of SSB (20,36). Other SSB interacting proteins (SIPs), such as *E. coli* RecQ, PriA and PriC, have been shown to influence the SSB-ssDNA binding mode, favoring the (SSB)_35_ mode (34,56,95) that occludes less ssDNA, thus making more ssDNA available for SIP binding. RecOR would then bind to the free ssDNA where RecR can function to stimulate RecA loading (33,77,81). Furthermore, SSB molecules that are tightly bound to DNA also must be displaced in order to load RecA onto ssDNA (33,69–73), as shown also in *D. radiodurans* and *B. subtilis* (75,76). A change in SSB binding mode to a less compact (SSB)_35_ would make more ssDNA available and facilitate RecA loading.

## DATA AVAILABILITY

The data underlying this article will be shared on reasonable request to the corresponding author.

## Supplementary Material

gkad084_Supplemental_File
