# Supplementary material for: Allosteric effects of E. coli SSB and RecR proteins on RecO protein binding to DNA
Source: Nucleic Acids Res. 2023 Feb 20;51(5):2284–97. doi: 10.1093/nar/gkad084 (PMC10018359; doi:10.1093/nar/gkad084)
Supplement: gkad084_Supplemental_File [file gkad084_supplemental_file.pdf]

Allosteric Effects of *E. coli* SSB and RecR on RecO Protein Binding to DNA
Supplementary Information

Min Kyung Shinn[1,2], Sumit K. Chaturvedi[1,3], Alexander G. Kozlov[1], Timothy M. Lohman[1]

[1]Department of Biochemistry and Molecular Biophysics, Washington University School of Medicine, St. Louis, MO 63130, United States
[2]Center for Biomolecular Condensates (CBC), Department of Biomedical Engineering, Washington University in St. Louis, St. Louis, MO 63130, United States
[3]Department of Biophysics, University of Delhi South Campus, New Delhi 110021, India

dsDNA annealing

Annealing of double-stranded (ds) DNA was confirmed with a native 10% polyacrylamide gel electrophoresis. The gel was pre-run at 15 W for 45 minutes before the samples were loaded. After the samples were loaded, the gel was run at 15 W for two hours in 1x TBE buffer.
ds18A: 5'-TGG CGA CGG CAG CGA GGC-3'
ds18B: 5'-Cy5-GCC TCG CTG CCG TCG CCA-3'
ds60A: 5'-CCA TGG CTC CTG AGC TAG CTG CAG TAG CCT AAA GGA TGA AAC TAG GAT CTT ATG CTC CAG-3'
ds60B: 5'-CTG GAG CAT AAG ATC CTA GTT TCA TCC TTT AGG CTA CTG CAG CTA GCT CAG GAG CCA TGG-3'

Fluorescence measurements

Experiments were performed using a PTI QM-2000 spectrofluorometer (Photon Technologies, Inc., Lawrenceville, NJ, USA) as previously described (1,2). 1.9 mL of ligand (RecO or ssDNA) in a 3 mL quartz cuvette were titrated with macromolecules (ssDNA or RecO). Samples were stirred throughout the experiments using magnetic stir bars inside the cuvettes. When monitoring Trp quenching of RecO with unlabeled oligo(dT), the excitation and emission wavelengths were set at 296 nm and 345 nm, respectively. When monitoring quenching of 5'-fluorescein-labeled oligo(dT), the excitation and emission wavelengths were set at 496 nm and 525 nm.

Oligonucleotide $(\varepsilon dA\text{-}dT)_L$ were synthesized with etheno(dA)-CE-phosphoramidite (εdA) (Glen Research, Sterling, VA, USA), such that a single εdA nucleotide was inserted every three (dT) nucleotides $(((dT)_3\varepsilon dA)_m(dT)_3$ for m = 3, 8, 17).Enhancement signals from εdA were monitored with the excitation and emission wavelengths at 276 nm and 405 nm.

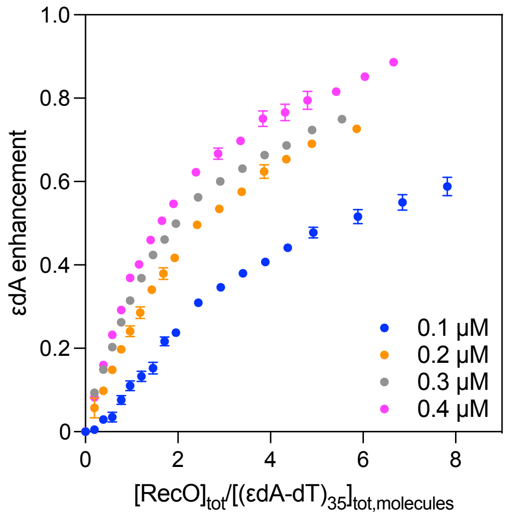

Figure S1 Fluorescence titrations of **ε**dA-$(dT)_{35}$ shows inconsistencies at different [ssDNA]. Titration of εdA-$(dT)_{35}$ with RecO while monitoring εdA enhancement at 0.1 μM (blue), 0.2 μM (orange), 0.3 μM (gray), and 0.4 μM (magenta). Titrations were performed while monitoring Trp quenching of intrinsic Trp residues of RecO, fluorescein quenching of 5'-labeled ssDNA, and εdA enhancement of εdA-$(dT)_L$. Similar to the titration of εdA-$(dT)_{71}$ in Fig. 1a, inconsistent extents of quenching is observed across different concentrations of εdA-$(dT)_{35}$.

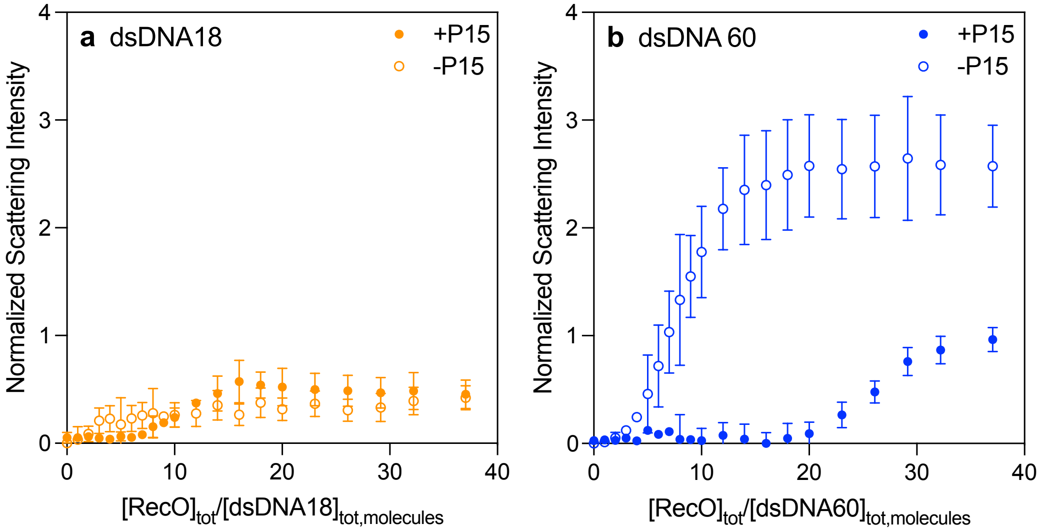

Figure S2 Light scattering is observed for dsDNA at larger molar excess of RecO to DNA compared to ssDNA. Titration of dsDNA (25 nM DNA molecules) that are (a) 18 bps and (b) 60 bps long with RecO while monitoring light scattering in the absence (empty circles) and presence (filled circles) of P15 (3.8 μM). (a) A small increase in light scattering intensity is observed both in the absence and presence of P15. This is in contrast to a ssDNA of similar length, $(dT)_{15}$, which did not show light scattering in the absence and presence of SSB-Ct peptides (Fig. 2a) but showed aggregate formation in confocal microscopy imaging (Fig. 3ai). (b) Light scattering is observed both in the absence and presence of SSB-Ct peptides in contrast to ssDNA substrates, where light scattering was significantly reduced or not observed in the presence of SSB-Ct peptides. The increase in scattering intensity, however, is observed at

a larger molar excess of RecO in the presence of SSB-Ct peptides (~25-fold excess) compared to in the absence (~3-fold excess).

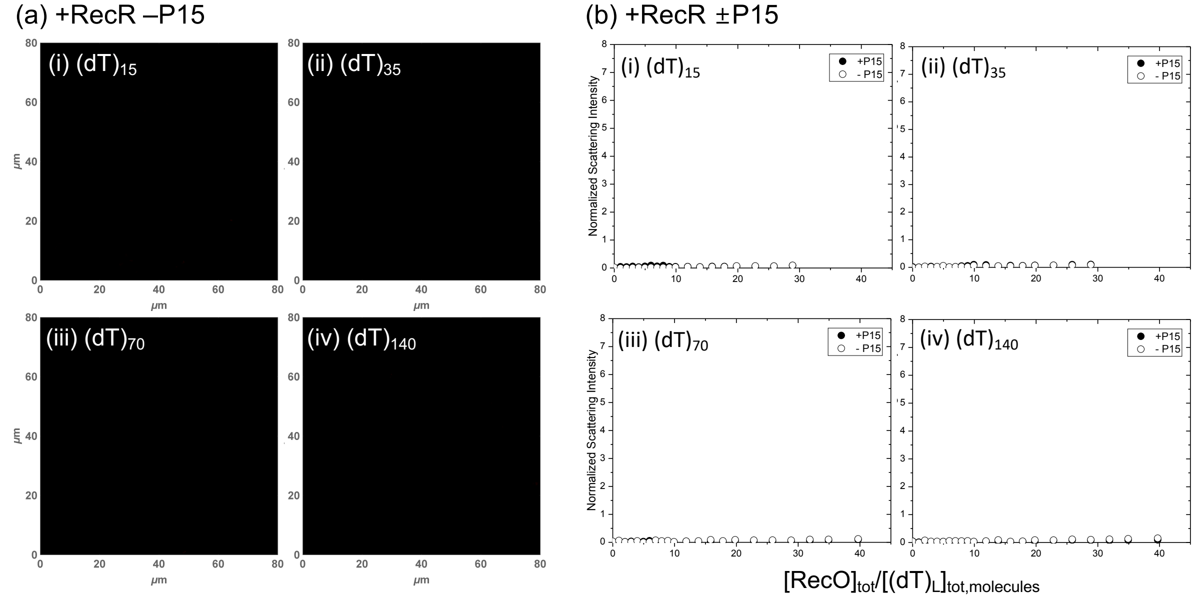

Figure S3 Aggregate formation is completely inhibited for RecOR-ssDNA complexes even in the absence of P15. (a) Confocal microscopy images of RecO (4 μM), RecR (16 μM), and (dT)$_L$(200 nM) mixtures for 3'-Cy3-labeled (i) (dT)$_{15}$ (ii) (dT)$_{35}$ (iii) (dT)$_{68}$ (iv) (dT)$_{140}$. At all lengths of oligonucleotides, aggregates are not observed, and only the black background is shown. This is in contrast to RecO-ssDNA complexes in the absence of RecR where amorphous aggregates were abundantly observed in the absence of P15. (b) Light scattering is not observed in the presence of RecR (2.52 μM) for titrations of and (dT)$_L$ (25 nM) with RecO (5 μM stock) in the absence (empty circles) and presence (filled circles) of P15 (3.8 μM) for (i) (dT)$_{15}$ (ii) (dT)$_{35}$ (iii) (dT)$_{70}$ (iv) (dT)$_{140}$. This is consistent with images shown in panel (a) which does not show any visible structures.

References

1. Kozlov, A.G., Galletto, R. and Lohman, T.M. (2012) SSB-DNA binding monitored by fluorescence intensity and anisotropy. *Methods Mol Biol*, **922**, 55-83.
2. Kozlov, A.G., Shinn, M.K., Weiland, E.A. and Lohman, T.M. (2017) Glutamate promotes SSB protein-protein Interactions via intrinsically disordered regions. *J Mol Biol*, **429**, 2790-2801.
3. Ryzhikov, M., Koroleva, O., Postnov, D., Tran, A. and Korolev, S. (2011) Mechanism of RecO recruitment to DNA by single-stranded DNA binding protein. *Nucleic Acids Res*, **39**, 6305-6314.
4. Shinn, M.K., Kozlov, A.G. and Lohman, T.M. (2021) Allosteric effects of SSB C-terminal tail on assembly of E. coli RecOR proteins. *Nucleic Acids Res*, **49**, 1987-2004.
